# Intensity modulated radiotherapy (IMRT) in bilateral retinoblastoma

**DOI:** 10.2478/v10019-010-0013-0

**Published:** 2010-09-09

**Authors:** Banu Atalar, Enis Ozyar, Kaan Gunduz, Gorkem Gungor

**Affiliations:** 1 Department of Radiation Oncology, Acibadem University Istanbul, Turkey; 2 Department of Ophthalmology, Ankara University Faculty of Medicine, Ankara, Turkey; 3 Department of Radiation Oncology, Acıbadem Maslak Hospital, Istanbul, Turkey

**Keywords:** retinoblastoma, radiotherapy, intensity modulated radiotherapy

## Abstract

**Background:**

External beam radiotherapy (EBRT) for retinoblastoma has traditionally been done with conventional radiotherapy techniques which resulted high doses to the surrounding normal tissues.

**Case report:**

A 20 month-old girl with group D bilateral retinoblastoma underwent intensity modulated radiotherapy (IMRT) to both eyes after failing chemoreduction and focal therapies including cryotherapy and transpupillary thermotherapy. In this report, we discuss the use of IMRT as a method for reducing doses to adjacent normal tissues while delivering therapeutic doses to the tumour tissues compared with 3-dimensional conformal radiotherapy (3DCRT). At one year follow-up, the patient remained free of any obvious radiation complications.

**Conclusions:**

Image guided IMRT provides better dose distribution than 3DCRT in retinoblastoma eyes, delivering the therapeutic dose to the tumours and minimizing adjacent tissue damage.

## Introduction

Retinoblastoma is the most common intraocular malignant tumour encountered in children. In most patients, retinoblastoma remains confined to the eye. However, in advanced cases, retinoblastoma can secondarily invade the orbit and metastasize to the central nervous system and other distant organs. Untreated retinoblastoma is nearly always fatal. Therefore, the early diagnosis and treatment is critical in saving lives of retinoblastoma patients and preserving a visual function of the affected eyes. Retinoblastoma occurs with an estimated frequency of 1/14000–1/34000 live births.^1^ In the United States, approximately 200 to 300 new cases are diagnosed each year. About 2/3 of the patients have unilateral and 1/3 have bilateral disease. More than 90% of the patients are diagnosed before the age of 5 years.^2^ Bilateral patients are generally discovered in the first year of life and unilateral ones are diagnosed later in the second year.^1^,^3^

Chemoreduction has changed the approach to the management of retinoblastoma. The dogma of enucleating the worse eye and irradiating the least affected eye in bilateral disease has largely been replaced by chemoreduction as a first step for both eyes. For the unilateral retinoblastoma chemoreduction is appropriate for those with Group A to C disease, but much less successful for those children with Group D or E retinoblastoma, which is usually treated by enucleation.

External beam radiation therapy (EBRT) is used less often today. It is used for moderately advanced tumours, multiple tumours, especially those with vitreous or subretinal seeds that fail chemoreduction. The external beam radiation dose is 35–45 Gy delivered over 4–5 weeks. An anterior lens-sparing, relative lens-sparing or modified lateral beam technique can be used. The anterior lens-sparing technique compared to the modified lateral beam technique leads to a higher tumour recurrence rate because the anterior retina is undertreated. On the other hand, the relative lens-sparing and modified lateral beam techniques yield similar eye conservation rates with subsequent salvage therapy. Much higher doses (from 50 Gy to 100 Gy) have been used in the past decades and it is quite possible that some second cancers have been due to the high radiation dose. The external beam radiation therapy can lead to significant complications such as facial hypoplasia from orbital bone atrophy, radiation cataract, and retinopathy.

The aim of this study was to compare the dose distribution of intensity modulated radiotherapy (IMRT) with the conventional external beam radiotherapy in terms of target and normal tissue doses in a recurrent bilateral retinoblastoma patient.

## Case report

An 8 month-old girl was referred to the Department of Ophthalmology, Ankara University Faculty of Medicine with the complaint of strabismus in the left eye. The examination under anaesthesia revealed bilateral group D retinoblastoma in both eyes. There was an exudative retinal detachment in both eyes with extensive subretinal seeds. There was no evidence of systemic involvement on bone marrow biopsy, spinal tap, and cranial MRI. The patient was initially treated with 6 cycles of intravenous carboplatin, etoposide and vincristine chemotherapy. Initially, the tumours in both eyes responded well to chemotherapy with resolution of SRF. The patient received several cryotherapy and transpupillary thermotherapy applications to recurrent and new tumours in both eyes over a period of approximately 12 months. However, the massive recurrence developed both eyes at 12 month follow-up and it was felt that either EBRT or enucleation was necessary at this point. The family opted for EBRT. The patient was seen in the Department of Radiation Oncology, Acibadem University, Istanbul for IMRT. A thermoplastic mask was prepared for the immobilization under anaesthesia and thereafter she underwent Computerized Tomography (CT) imaging with 1-mm slices for treatment planning purposes. Target tumour volumes and organs at risk (OAR) such as orbital bone, cornea, lens, lacrimal gland and optic nerve were delineated.

Gross tumour volume (GTV)^4^ dose was not specified in this case, only the recurrent tumours in both eyes were delineated as tumour in order to not to lower the dose in those areas; clinical tumour volume (CTV) was defined as both right and left retina and planning target volume (PTV) was generated from CTV plus 1 mm margin. Dose to OAR was defined according to previously reported data.^5^–^9^

## Comparison of 3DCRT and IMRT

**FIGURE 1:**
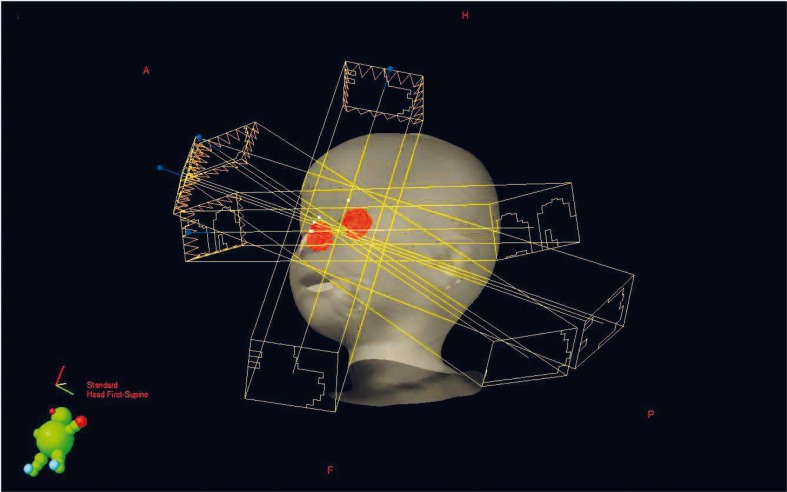
Four-field noncoplanar IMRT plan

In order to provide dose constraints for OAR we performed 4 different IMRT plans and a conformal plan. Of these IMRT plans the best isodose distribution and the dose volume histogram were provided with a noncoplanar 4-field technique (Figure 1); when compared to a conformal plan there was no significant difference for cornea, lens and optic nerve doses. The patient was treated with 4-field noncoplanar IMRT plan to a total dose of 40 Gy, 2 Gy per fraction under general anaesthesia. According to our department’s image guided radiotherapy (IGRT) protocol, daily kilovoltage images were taken from anteroposterior and lateral fields before each treatment and corrections were done by matching pretreatment images with digitally reconstructed radiographs.

Radiation doses to the orbital bones and lacrimal glands were apparently lower while the tumour dose was higher in the IMRT plan. As a result of using multiple non coplanar beams; there were low dose areas in brain, brainstem and hypophysis with IMRT plan whereas no dose with 3DCRT, but these doses were below 5 Gy which was a safe dose for the affected areas. The comparison of doses between conformal and IMRT plan is detailed in Table 1.

At one year follow-up, the patient remained free of any obvious radiation complications.

## Discussion

Retinoblastoma is a radiosensitive tumour. There is a wide spectrum of techniques used for retinoblastoma ranging from single fields to complex fields such as anterior lens sparing technique, lateral oblique fields, multiple non coplanar arcs, single anterior electron fields, stereotactic radiotherapy, conformal and intensity modulated radiotherapy plans. Even protons were used to perform homogeneous dose coverage of retina while sparing the lens and bony anatomy.^5^,^10^–^13^ IMRT for retinoblastoma was first reported by Krasin *et al*.^5^ Subsequently, Reisner *et al.* published a comparative analysis of external radiotherapy techniques with IMRT in a case report of unilateral retinoblastoma.^8^ Previous reports on IMRT planning for retinoblastoma revealed greater sparing of the surrounding bony orbit and lacrimal gland as in our study.

High doses affecting bony orbital structures may cause growth arrest of orbital fossa and facial asymmetry.^7^ IMRT leads to lower doses in orbital bones, while not reducing retinal doses. In our bilateral IMRT plan, doses in both orbital bones were higher when compared to unilateral cases of Reisner *et al*.^8^ These relatively high doses can be explained by the location of recurrent tumours; which were in the posterior poles of both eyes. Plans were done in order to have an optimal dose in these regions. In cases where the tumour is located medially or anteriorly, a lower dose may be delivered to the orbital bones using IMRT.

Dry-eye syndrome, because of lacrimal gland exposure to radiation, is also another important and irreversible complication for this patient group threatening life quality. One of the main advantages of IMRT is to reduce lacrimal gland dose without lowering retinal doses. Our patient’s mean lacrimal gland doses were less than 30Gy. Dry eye is quite unlikely to develop with these radiation doses as reported by Parson *et al*.^6^

The optic nerve is also affected in the radiotherapy of retinoblastoma. Doses exceeding 54 Gy may lead to the development of radiation optic neuropathy leading to irreversible visual impairment. Reisner *et al.* reported maximum doses as high as 48 Gy for the optic nerve dose with several techniques including their IMRT planning.^8^ In our setting the optic nerve received a maximum dose of 40–41 Gy which is a safe dose for optic neuropathy. The reduction in the optic nerve dose may prevent visual problems in the future life of the patient.

Corneal injury after EBRT has also been reported previously. The critical dosage was considered 50 Gy as the 50% risk at 5 years for cornea.^14^ Reisner *et al.* considered V26.5 for the evaluation of corneal injury probability based on the study of Jiang *et al*.^9^,^10^ Our plan delivered less than 50 Gy to the cornea region (mean dose for right and left cornea was 32 Gy and 28.7 Gy respectively) but the V26.5 dose was relatively higher especially on the right side, where a tumour was located more anteriorly.

The lens is the most radiosensitive tissue in the eye.^15^ Lens preservation was always been an important target in radiotherapy planning for the treatment of tumours around the eye region. Doses exceeding 12 Gy usually results cataract. Lens sparing techniques with EBRT also caused cataract in 28% of patients.^16^ However, a good outcome after the cataract surgery with phacoemulsification was reported even in young ages.^17^ Therefore, we preferred to achieve therapeutic doses in the entire to avoid the recurrence of the tumour rather than delivering subtherapeutic doses to the retina in an effort to preserve the lens from the cataract development.

Technologic developments improved outcomes enormously in the last 10 years for EBRT. The capability of protecting normal tissue around tumour became available. IMRT with image guidance, so called IGRT-IMRT, is the superior technique that allows us to do the best and safe treatment. Outcomes of IMRT were successful with the more common cancers including prostate, head and neck, breast cancers in terms of the increased local control and normal tissue protection. Even with lung cancer, where a significant organ and tumour movement may be a problem in radiotherapy, IMRT proved to be successful. The outcomes of IMRT in rarer tumours such as retinoblastoma are not widely known because of the paucity of publications in this area.

It has been concluded that any genotoxic therapy can induce second neoplasms after long latent times and the risk is slightly higher with radiotherapy but the side effects of radiotherapy have less impact on the patients’ quality of life when compared with other therapies.^18^ In the pediatric setting the risk could be significant due to a higher inherent susceptibility of tissues. However, as the risk of secondary cancers as sarcomas, related with IMRT estimated to be 2% compared with 1% for 3DCRT, the use of protons became actual to reduce risk of radiation-induced carcinogenesis.^19^ The efficacy of IMRT in reducing the acute and late toxicity in children with nasopharyngeal carcinoma (NPC) was reported by two centres recently.^20^,^21^ Louis *et al.* found no difference with IMRT in terms of late toxicity such as hypothyroidism, xerostomia, hearing loss, and dental disease.^20^ On the other hand Laskar *et al.* concluded that IMRT significantly reduces and delays the onset of the acute toxicity compared to EBRT, resulting in the improved tolerance and treatment compliance for children with NPC.^21^ However, the number of studies with IMRT in pediatric tumours was very limited and other centre experience should be awaited.

In conclusion; image guided IMRT provides better dose distribution than 3DCRT in retinoblastoma eyes, delivering the therapeutic dose to the tumours and minimizing adjacent tissue damage. In terms of avoiding radiation complications including dry eye syndrome, facial deformity, cataract, radiation retinopathy and radiation papillopathy, IMRT planning should always be taken into consideration for patients that are referred for radiotherapy.

## Figures and Tables

**Table 1:** Comparison of doses for 3DCRT and IMRT plan of our patient

	**3DCRT**			**IMRT**		
	**Mean (cGy)**	**Max. (cGy)**	**Vol/dose**	**Mean (cGy)**	**Max. (cGy)**	**Vol/dose**
**R. Lens**	3304	3676		2763	3134	
**L. Lens**	2657	3299		2639	3270	
**R. Cornea**	3228		V26.5<78%	2609		V26.5<70.6%
**L. Cornea**	2874		V26.5<67%	2909		V26.5<50.6%
**R. Optic nerve**		3830			4147	
**L. Optic nerve**		3841			4063	
**R. Lac. Gland**	3807		V34<99%	2527		V34<5%
**L. Lac. Gland**	3741		V34<100%	2456		V34<0%
**Orbital Bones**	2204		V20<56.8%	1965		V20<49.7%

V20 (volume received above 20 Gy), V34 (volume received above 34 Gy), V26.5 (volume received above 26.5 Gy)
